# Pathological and Therapeutic Significance of Tumor-Derived Extracellular Vesicles in Cancer Cell Migration and Metastasis

**DOI:** 10.3390/cancers15184425

**Published:** 2023-09-05

**Authors:** Giovanna L. Liguori, Veronika Kralj-Iglič

**Affiliations:** 1 Institute of Genetics and Biophysics (IGB) “Adriano Buzzati-Traverso”, National Research Council (CNR) of Italy, 80131 Naples, Italy; 2 University of Ljubljana, Faculty of Health Sciences, Laboratory of Clinical Biophysics, SI-1000 Ljubljana, Slovenia; veronika.iglic@fe.uni-lj.si

**Keywords:** extracellular vesicles, tumor-derived extracellular vesicles, cancer cell migration, epithelial-mesenchymal transition, tumor metastasis, cancer therapy, alternative signaling routes, drug delivery, extracellular vesicle exploitation, quality control

## Abstract

**Simple Summary:**

Cancer metastasis accounts for almost 90% of cancer deaths worldwide. The efficiency of current surgical, radiotherapeutic, and chemotherapeutic approaches is limited, and new, more powerful therapeutic strategies are urgently needed. Tumor metastasis as well as primary tumor invasiveness strongly rely on the ability of cancer cells to migrate at distant sites or locally in the surrounding tissue. Here, we give an overview of the underlying mechanisms, with special emphasis on the role of extracellular vesicles (EVs). EVs are nanoparticles released by cells implied in cell–cell communication. They can travel in the bloodstream and in other body fluids, thus transporting molecules to specific cells and inducing specific responses. Based on the recent literature, we discuss the role of vesicles in cancer cell migration and metastasis as well as their anti-cancer therapeutic significance, focusing on tumor-derived EVs. Moreover, we propose EV encapsulation as an alternative route with specific effects on target cells.

**Abstract:**

The infiltration of primary tumors and metastasis formation at distant sites strongly impact the prognosis and the quality of life of cancer patients. Current therapies including surgery, radiotherapy, and chemotherapy are limited in targeting the complex cell migration mechanisms responsible for cancer cell invasiveness and metastasis. A better understanding of these mechanisms and the development of new therapies are urgently needed. Extracellular vesicles (EVs) are lipid-enveloped particles involved in inter-tissue and inter-cell communication. This review article focuses on the impact of EVs released by tumor cells, specifically on cancer cell migration and metastasis. We first introduce cell migration processes and EV subtypes, and we give an overview of how tumor-derived EVs (TDEVs) may impact cancer cell migration. Then, we discuss ongoing EV-based cancer therapeutic approaches, including the inhibition of general EV-related mechanisms as well as the use of EVs for anti-cancer drug delivery, focusing on the harnessing of TDEVs. We propose a protein-EV shuttle as a route alternative to secretion or cell membrane binding, influencing downstream signaling and the final effect on target cells, with strong implications in tumorigenesis. Finally, we highlight the pitfalls and limitations of therapeutic EV exploitation that must be overcome to realize the promise of EVs for cancer therapy.

## 1. Introduction

The degree of tumor infiltration and the formation of metastases are key aspects in the prognosis of patients with cancer diseases. Current treatment strategies such as surgery, radiotherapy, and chemotherapy, even in combination, have a limited effect on metastatic cancers; hence, cancer metastasis accounts for almost 90% of cancer deaths worldwide [1]. Therefore, there is an urgent need to develop more powerful therapeutic approaches targeting the complex mechanisms regulating cancer cell invasiveness and metastasis. Both these features rely on the ability of cancer cells to move and to migrate locally or at distant sites. Cancer cell migration is a central biological process in cancer pathogenesis, whose targeting represents an ambitious as well as desirable goal to prevent tumor spreading and metastasis. To manage this hallmark of cancer progression, a deep understanding of the mechanisms of cell migration and its regulatory signals is therefore fundamental.

Even though cell migration has been extensively studied and the main steps involved identified, many aspects still need to be unraveled. Cell migration depends on complex relationships with the surrounding cells and with the extracellular space. It can be single or collective and involves an orchestrated and finely tuned series of intracellular cytoskeletal as well as extracellular adhesion rearrangements [2,3]. The exchange of information among cells is attained through the release of specific soluble or immobilized signaling molecules and their interaction with corresponding receptors, or through direct cell-to-cell communication that includes gap junctions, cytonemes, tunneling nanotubes and extracellular vesicles (EVs) [4,5,6,7,8]. With cancer cell migration being based on and regulated by local and distant communication among cells, EVs, as important mediators of cell communication, play a strategic role in regulating the migration of cancer cells. Thanks to their ability to travel long distances, be transported into body fluids, and interact with specific target cells, EVs are deeply involved in cancerogenesis at multiple steps. EVs contribute to the regulation of cancer states during epithelial-mesenchymal transition (EMT) and its reverse, mesenchymal-epithelial transition (MET), as well as to cancer invasion, immune escape and metastasis at target sites [1,9,10,11,12]. Through EV production, transport and uptake, several proteins, lipids and nucleic acids with oncogenic properties can be transferred between cells. Therefore, EVs are considered new and promising sources of both diagnostic and prognostic biomarkers as well as therapeutic targets for a variety of cancer types, including pancreatic, ovarian, prostate, breast, colorectal cancer and glioblastoma multiforme (GBM) [13,14]. Moreover, due to their low immunogenicity, EVs may cross interspecies boundaries and are extremely suitable for the targeted delivery of anti-cancer drugs and the development of therapeutic applications to counteract cancer progression and tumor metastasis [15,16,17,18,19].

In this review, we summarize the main features of cancer cell migration and the contribution of tumor-derived EVs (TDEVs) in regulating this cancer hallmark. Moreover, we present the main anti-cancer therapeutic strategies that are actually under implementation, focusing on the harnessing of TDEVs for cancer therapeutic drug delivery but also as therapeutic agents *per se*. Related to this, we hypothesize that, besides secretion or cell membrane binding, a protein-EV shuttle might serve as an alternative route and signaling pathway able to induce a specific effect on target cells, different from the one induced by soluble or membrane-bound forms, with strong implications in tumorigenesis.

## 2. Migration of Cancer Cells: Why, What, How, and When

Cell migration is an essential process for life and development as well as an integral mechanism of many pathological processes, including tumor cell dissemination [20]. Non-migratory tumor cells need to be stimulated to migrate, and invading tumor cells alter their behavior in response to cues received from the extracellular environment. Stimuli can be biochemical, represented by soluble factors and chemoattractants (chemotaxis), or, as more recently highlighted, biophysical, including the molecular composition (haptotaxis), the microarchitecture, the thickness and the stiffness (durotaxis) of the extracellular matrix (ECM) that surrounds cells, as well as the electric fields generated in the local environment (galvanotaxis) [20,21]. Finally, cell motility is strongly influenced by the geometry and the topography (topotaxis) of the substrate and the curvature of the surface (curvotaxis) on which cells move [22]. The manifold mechanisms underlying the complexity of the interaction between cells with their surrounding matrix are also summarized under the term “materiomics” [23].

In all cases, stimuli must be internalized to produce the cellular rearrangements and cytoskeletal remodeling able to confer cell polarization and migratory behavior. Signal internalization is mediated by transmembrane receptors on the cell surface, such as enzyme-linked, G protein-coupled, and ion channel-linked receptors, as well as integrins, whose activation triggers signaling pathway cascades inside the cell [2,21]. Transmembrane receptors are usually distributed heterogeneously among cells. Moreover, the cell membrane contains different actively maintained micro- and nanodomains, such as ruffles, lipid rafts, microvilli, or caveolae, characterized by different protein and lipid compositions as well as receptor profiles. Asymmetry in protein and lipid composition confers to the plasmatic membrane its own fluidity and spontaneous curvature [24]. Upon binding to the ligand, the activation of receptor tyrosine kinases (RTKs) induces cytoskeletal organization and also changes in gene expression, culminating in changes of cell polarity [2]. Polarity is regulated by the coordinated interactions of short-range positive and long-range negative feedback loops [25,26,27,28,29]. When migratory cells first sense external stimulatory cues, they rapidly activate leading-edge polarity factors throughout the plasma membrane [27,30], resulting in actin filament polymerization and the formation of multiple sheet-like protrusions [29,31]. In parallel with this initial step of activation, cells generate long-range negative feedback (e.g., growth of actin filaments) [25] to enable a dominant front to emerge [31,32].

It was recently found that a high concentration of curved proteins may induce protrusive forces and sheet-like lamellipodia on highly curved surfaces in order to minimize membrane free energy [33,34]. Theoretical considerations show that the redistribution of membrane proteins and active forces are closely connected to the membrane shape [35]. Integrins function as biomechanical sensors of the microenvironment and are able to interact with specific components of the ECM and sense the ECM forces [36]. Upon activation, integrin clustering initiates the formation of adhesion complexes, recruiting different proteins, such as Tensin-, Talin-, Vinculin-, and Actin-related proteins, which induce protrusion formation, as well as focal adhesion kinase (FAK), with the induction of downstream signaling that controls cell adhesion and motility. Interestingly, integrin function is also mediated by the crosstalk with RTKs and other coreceptors, which can enhance growth-factor receptor activation and FAK phosphorylation [37]. Mechanical stimuli are also able to activate growth factor receptors independently of their ligands, and the dynamics of these processes allow for adhesion turnover, which is essential for mesenchymal cell migration [38].

**Figure 1:**
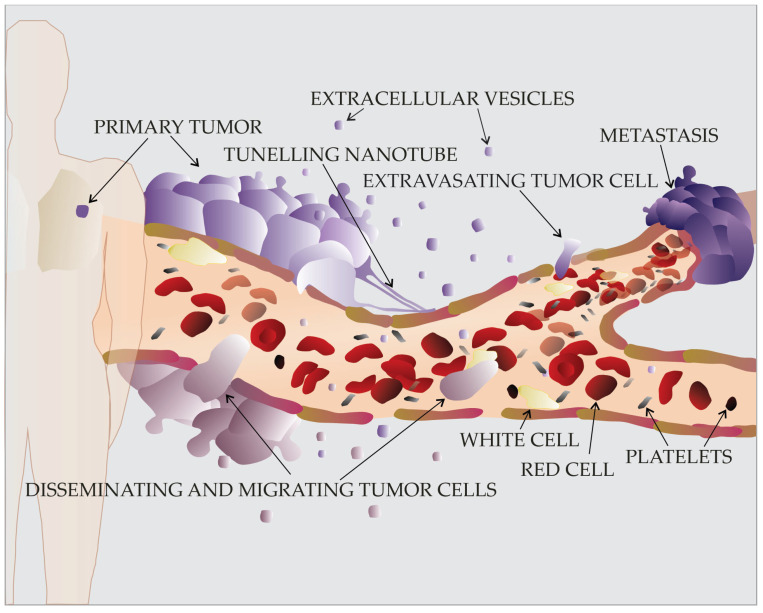
Tumor cell migration in blood circulation and formation of metastasis at distant sites.

There is also ample evidence that substrate stiffness plays a role in cancer metastasis. Increased stiffness may hinder cell migration due to an excessive steric hindrance, whereas decreased cell stiffness promotes the detachment and migration of tumor cells [39]. In fact, one of the first steps for cancer cells to acquire migratory abilities is the release of proteases, including matrix metalloproteases (MMPs) and other lytic enzymes that are able to digest ECM. Cancerized ECM undergoes extensive and dynamic reorganization, decreasing its stiffness, which in turn promotes cancer cell migration. In response to decreasing matrix rigidity, RhoA GTPase is activated in the cells, thereby inducing a transition toward a mesenchymal phenotype through a complex and reversible cellular transformation such as EMT. EMT is characterized by intense cytoskeletal rearrangements, the loss of E-cadherin, increased expression of fibronectin and vimentin, increased cell rounding, and the acquisition of an amoeboid phenotype [40,41]. The different stages of EMT, the so-called EMT continuum, and its reverse process, MET, are deeply involved in the phenotypic heterogeneity of cancer cells, which contributes to hallmark features of cancer such as tumor invasion, metastasis formation, and development of chemoresistance [42]. After EMT, tumor cells may disseminate within the body with the circulation of blood (Figure 1). There, they are subjected to vascular routing, collisions and associations with blood cells, hemodynamic shear forces, and physical constraints imposed by the vessel architecture [43,44,45]. If the disseminated cancer cells are arrested and if they overcome hostile shear forces exerted by the surrounding tissue [46,47], they may find a position favorable for adhesion and subsequent metastasis formation [48].

## 3. Extracellular Vesicles: A Short Overview

EVs have been extensively studied and described as micro- and nanoparticles that are delimited by a lipidic bilayer, released by most cells, full of cellular content, and have functional relevance. EVs can target cells through their interaction with specific cell membrane molecules and induce specific responses through the direct activation of signaling receptors and their downstream cascades or by releasing their cargo inside the cells. EV cargo includes proteins, lipids, metabolites, and nucleic acids (DNA, microRNA, long non-coding RNA, circular RNA), which are able to cause changes in gene expression or protein interaction. The horizontal transfer of cellular components is crucial for transferring functional properties from the cells of origin to the recipient cells and/or to influence the phenotypic, morphological, and functional features of the target cells and possibly force them to create a microenvironment beneficial to the cells of origin [8,11,49,50].

**Figure 2:**
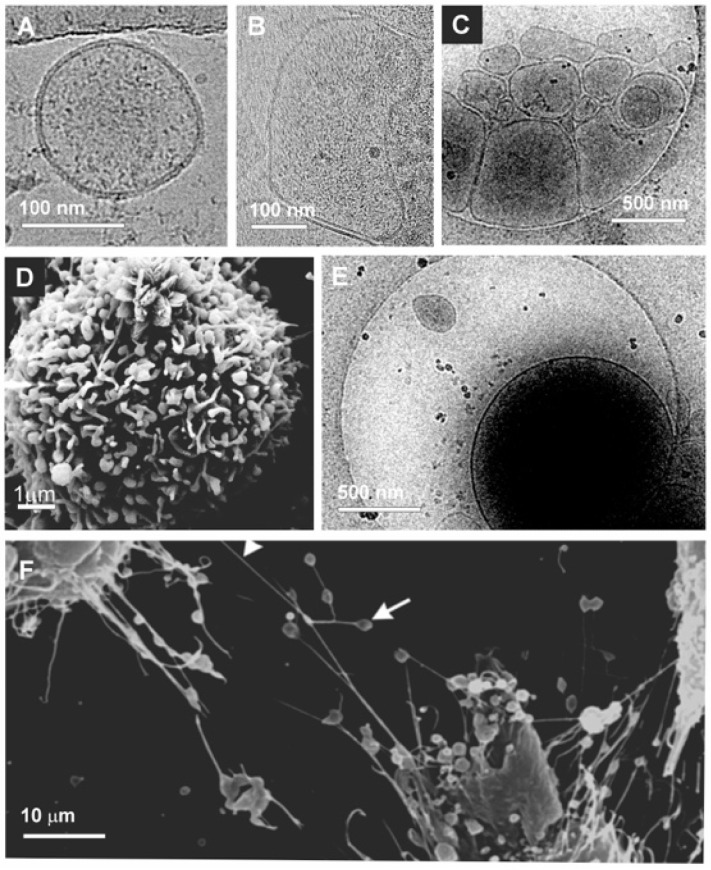
Electron microscopy images of extracellular vesicles and tunneling nanotubules formed by tumor cells. (**A**–**C**) Cryogenic electron microscopy (Cryo-EM) images of small (**A**) and large (**B**,**C**) extracellular vesicles (EVs) isolated from human teratocarcinoma cells. (**D**) Scanning electron microscopy (SEM) image of EVs budding from the membrane of human bladder cancer cell. (**E**) Cryo-EM images of particularly large vesicles or oncosomes released by human teratocarcinoma cells. (**F**) SEM images of tunneling nanotubes (white arrowhead) with dilatations that may act as gondolae (white arrow) in the transport of matter between neighboring urothelial cancer cells [53].

Based on their size and density, EVs have been subdivided into small and medium–large EVs, whose diameter is approximately 30–200 nm and 200–1000 nm, respectively (Figure 2A–C). Based on biogenesis, scientists identified small vesicles that originated through the endocytic pathway, which were named exosomes—the most popular term in the EV field. EVs originated through blebbing and successive budding from the plasmatic membrane (Figure 2D) were named ectosomes or microvesicles. The latter, escaping from size restriction due to the endocytosis/exocytosis pathway that characterizes exosomes, are more heterogeneous in size, being as small as exosomes and reaching 1 μm in diameter [8,11,51,52].

In the context of cancer cell migration, we aim to also outline two other specific types of vesicles, which are (large) oncosomes and migrasomes. Oncosomes, or large oncosomes, (LOs) are extremely large EVs with a diameter of 1–10 μm, budding specifically from cancer cells (Figure 2E). The term oncosome was introduced by Al-Nedawi and coworkers in 2008 to indicate large EVs released from glioma cells that were capable of transferring the oncoprotein epidermal growth-factor receptor variant III (EGFRvIII) to the membrane of tumor cells lacking this receptor, thus propagating tumor-promoting content and inducing the transformation of the targeting cell [54]. Then, LOs with an even greater size were found to be released by prostate cancer cells [55]. Oncosomes probably share some features with MVs because of their common blebbing origin from the membrane, but probably due to their large size and their specific tumor derivation, they show specific characteristics that make them key players in tumor progression and metastasis formation [56,57,58].

On the other hand, migrasomes are recently discovered organelles produced by migrating cells [59]. During their movement, migrating cells leave long tubular strands, named retraction fibers, behind them, showing large EVs at their tips and intersections, often containing smaller vesicles. When retraction fibers, which connect EVs with the main cell body, break, migrasomes are released in the extracellular space [59,60]. Migrasomes can rupture and release their luminal contents into the environment by a process named “migracytosis,” or they can be taken up by the surrounding cells and transfer their content into them. Migrasomes can be produced by several cancer cells, such as colon, breast, ovarian, gastric and pancreatic cancer cells [61], and can modulate tumor cell proliferation via laterally transferring oncogenic mRNA and protein [62]. It is also conceivable that migrasomes derived by cancer cells may promote tumor aggressiveness and metastasis formation [63]. However, the study of migrasome function is still in its infancy, and it would be very interesting to focus future investigations on this aspect.

Finally, related to EVs, the tunneling nanotubes (Figure 2F) are also considered another type of cell communication, allowing for the rapid exchange of cellular cargos between connected non-adjacent cells, including organelles, vesicles, molecules, ions and pathogens [64]. Originally, a network connecting giant phospholipid vesicles was predicted by Mathivet and coauthors (1996) [65], and the existence of stable nanotubes composed of phospholipid membranes that could constitute such a network was experimentally proved [66,67,68,69]. Later, such structures were also observed in cells [35,70]. Interestingly, in both giant phospholipid vesicles and in cells, tubes showed dilatations that looked like gondolae (Figure 2F). In giant unilamellar lipid vesicles, the gondolae moved along the tube, and in the final stages of such transport, the bleb was fused with the cell membrane and released the contents into the vesicle [5]. It is tempting to speculate that, as in the case of migrasomes, vesicular structures could derive from gondolae after breaking tunneling nanotubes.

## 4. Tumor-Derived Extracellular Vesicles in Cancer Cell Migration and Metastasis

### 4.1. Increase of Extracellular Vesicle Release in Migrating Cancer Cells

Although EV measurements in body fluids are still hampered by heterogeneity in both the collection of samples and the methods of isolation and counting, clinical studies indicated that total EV concentration in blood samples is a potential indicator of clinical status. EV counting, then, might be used to follow the development of a cancer disease as well as for predicting and monitoring tumor response to therapy [71,72]. Moreover, EVs released by cancer cells are usually more numerous and molecularly different than those derived from the normal counterpart, and the number and content of TDEVs depend on the tumor of origin, the state of the disease, and the response to therapy [11,73]. In agreement, an increase in tunneling nanotube numbers was also associated with malignant transformation, as demonstrated by comparative studies in normal and malignant invasive urothelial cell lines [74].

The acquisition of migration and invasion abilities is accompanied by increased vesicle shedding from the membrane. Through the induction of EGFR coupled with the blockade of E-cadherin, both human colon epithelial cancer and squamous carcinoma cells were shown to acquire mesenchymal features and release a higher number of EVs [75]. In invading tumor cells, the small GTPase RhoA is activated in response to decreased ECM stiffness and promotes a shift towards a more amoeboid phenotype and the release of abundant tumor MVs from the membrane [76]. Additionally, ezrin phosphorylation downstream of RhoA signaling also causes an increase in tumor MV shedding [77]. Interestingly, sites of active MV release serve as convergence points of multiple intracellular trafficking pathways, of which many components are subsequently loaded in tumor MVs, such as the Ras-related GTPases RhoA, Rab22a, and Rab35, and the ADP-ribosylation factor 6 (ARF6) [73]. ARF6, in particular, is involved in the regulation of actomyosin contractile machinery, but also ARF6-positive endosomes serve as a repository for tumor MV-specific cargo, such as β1-integrin and membrane type 1 (MT1)-MMP [78]. MVs are loaded with several proteases and are then fully capable of degrading ECM, in turn promoting amoeboid cell invasion [78]. Among proteases, MT1-MMP has a key role in facilitating tumor cell invasion in collagen-rich matrixes [79]. The depletion of MT1-MMP in tumor MVs strongly reduces the invasive capacity of melanoma cells, without affecting MV shedding and other MV content [80].

Exosomes have been also implicated in directional motility and the invasion of tumor cells. Live confocal imaging of fibrosarcoma cells revealed exosome secretion at the leading edge of migrating cells [81]. Moreover, invadopodia at the adherent cell surface are supposed to serve as docking sites for the multivesicular bodies (MVBs) implied in exosome formation and release [82]. Functional studies in which MVB biogenesis was impaired through Rab27a knockdown evidenced migration defects in both fibrosarcoma and squamous carcinoma cells [83]. Like MVs released from cancer cells, exosomes formed in correspondence with invadopodia are full of proteases and are deeply involved in ECM degradation and remodeling [73].

### 4.2. Functional Implications of Tumor-Derived Extracellular Vesicle Cargo

It is now well established that cargo from EVs released by highly metastatic cancer cells is significantly different compared to that of non-metastatic cancer cells [84,85]. Several studies on cells of various cancer types produced a plethora of evidence on the EV-mediated transfer of mesenchymal features, migration capabilities and resulting metastatic potential [1,86,87]. EVs from bladder cancer cells were able to induce the migration of urothelial cells by downregulating the expression of the epithelial markers E-cadherin and β-catenin and inducing the expression of mesenchymal genes (alpha-smooth muscle actin or α-SMA and S100 calcium-binding protein A4 or S100A4) [88]. Moreover, after exposure to EVs released by highly metastatic hepatocellular carcinoma cells, low metastatic hepatocellular cancer cells underwent an EMT, with the acquirement of migration and invasiveness abilities [89]. Similarly, EVs collected from the serum of late-lung-cancer patients induced an EMT in normal bronchial epithelial cells, which led to the acquisition of migration, invasion, and proliferation abilities [90]. EVs from human bladder cancer patients also induced the expression of mesenchymal genes in their normal counterparts [88]. Interestingly, *in vivo*, the injection of poorly metastatic B16-F1 melanoma cells pre-treated with B16 aggressive melanoma cell-derived EVs caused lung metastasis in injected mice [91].

**Figure 3:**
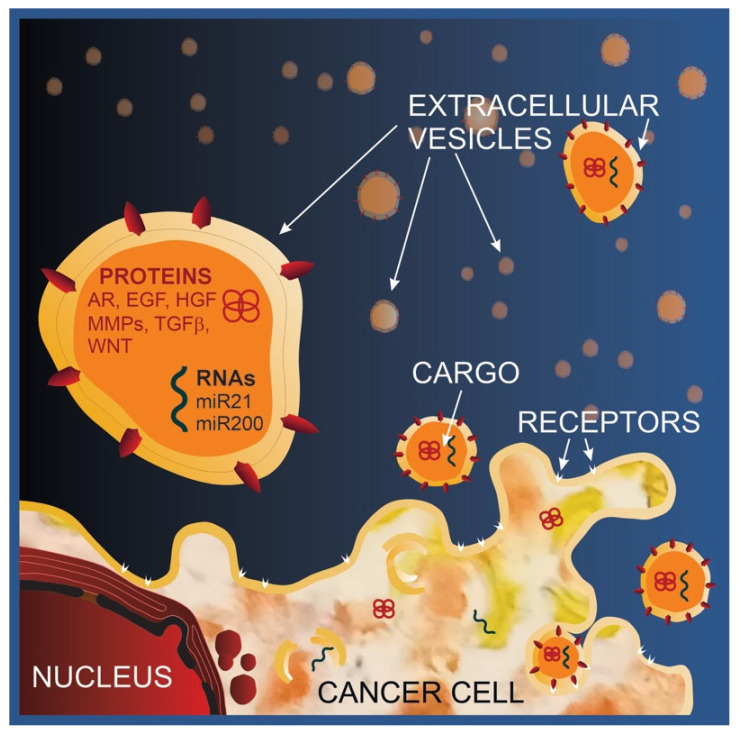
EV functional cargo inducing mesenchymal features and migratory abilities in cancer cells. Abbreviations: AR, amphiregulin; EGF, epidermal growth factor; HGF, hematopoietic growth factor; MMPs, metalloproteases; TGFβ, transforming growth factor β; WNT wingless-related integration site.

Research studies then focused on the identification of the signaling molecules involved in the metastatic shift of recipient cells, some of which are reported in Figure 3.

EGFR ligands, such as amphiregulin, were found in EVs produced by both human breast and colorectal cancer cells and were able to increase the invasiveness of recipient cancer cells [92]. The transforming growth factor β (TGFβ), wingless-related integration site (WNT), epidermal growth factor (EGF), hematopoietic growth factor (HGF) and MMPs, all involved in EMT, motility and invasiveness promotion, were all found to be associated with TDEVs [93]. Besides proteins, pro-metastatic nucleic acids, and in particular microRNAs, have also been identified [94,95,96]. As an example, miR-21 is enriched in TDEVs that significantly promote the proliferation and metastasis of breast cancer cells through EMT induction [97]. miR-200, which regulates the expression of the EMT-inducing transcription factors zinc finger E-box-binding homeobox 1 and 2 (Zeb1 and Zeb2), was shown to be transferred via EVs from metastatic to non-metastatic breast cancer cells, where it was able to induce EMT. Interestingly, EVs released from miR-200-expressing tumors transferred the ability to colonize distant sites and form metastasis to low metastatic cells [98].

All cancer cells exhibit specific tropisms to predetermined sites or organs where they metastasize. Cancer spreading to specific sites was first described in 1989, in the “seed and soil” hypothesis of Paget, according to whom specific cancer cells entering into the blood circulation (the seeds) could extravasate and adhere to specific organs presenting a favorable environment for dissemination and tumor growth (the soil) [99,100]. TDEVs may act as fertilizers in the seed and soil hypothesis by conditioning normal niche cells at sites distant from the primary tumor to adopt a pre-metastatic phenotype, attracting the arrival of cancer cells as well as aiding in the growth of secondary tumors [17,101,102]. MET-containing melanoma-derived EVs, for instance, are able to induce a pre-metastatic state in bone marrow progenitor cells [102]. Similarly, EVs released by pancreatic cancer cells were shown to prime the liver for metastasis development through the transfer of macrophage migration inhibitory factor contained within [103]. Among the different molecules associated with this phenomenon, integrins are the TDEV cargo mainly responsible for organotropism during the process of tumor metastasis [104]. Integrin αV, for instance, was demonstrated to promote the formation of a pre-metastatic niche that facilitates bone colonization by breast cancer cells [105]. Integrin αV is also contained in LOs released by prostate cancer cells and is responsible for the induction of migration in dermal and tumor endothelial cells and for the increased adhesion and invasion of recipient cells through the activation of AKT and increased MMP expression [106,107]. Integrins are also required to establish the adhesion with the ECM along retraction fibers, then acting as platforms for migrasome biogenesis [108]. Moreover, EVs from brain-tropic metastatic cancer cells carrying cell migration-inducing and hyaluronan-binding protein (CEMIP) target brain endothelial and microglial cells, leading to brain vascular remodeling and metastasis [109]. Finally, EVs released by melanoma cells downregulate type I interferon receptor and cholesterol 25-hydroxylase, thus facilitating EV uptake by normal cells and further establishing a pre-metastatic niche [110]. Not only proteins but also RNAs have been shown to be implicated in pre-metastatic niche conditioning. EVs released by metastatic breast cancer cells carrying miR-105 cause a tight junction decrease in endothelial cells in correspondence with various organs, with a consequent systemic vascular leakiness promoting the extravasation of tumor cells and the formation of distant metastasis [111]. In addition, EVs carrying miR-181c derived from brain-metastatic breast cancer cells trigger the breakdown of the blood–brain barrier, thereby promoting cancer cells’ access to the brain [112].

## 5. Extracellular Vesicle Significance in Targeting Cancer Development and Metastasis

### 5.1. Extracellular Vesicles as Therapeutic Targets

TDEVs have been implicated in several pro-metastatic mechanisms. Therefore, EV-based cancer therapeutic approaches focused on impairing TDEV release, transportation on route and uptake as means to reduce metastasis, as summarized in Table 1.

A major challenge for these types of strategies is how to specifically block TDEV biogenesis, transportation and uptake without affecting normal EV pathways. To address this issue, extensive basic research on genetic and cellular pathways implicated in the production/uptake of various types of EVs is taking place. Besides these studies, high-throughput screening of pharmacological compounds in both cancer and normal cells is ongoing to identify potential effectors for cancer metastasis-specific targeting. Although EV pathways may be shared by cancer and non-cancer cells, it is conceivable that those involving TDEVs, or a precise subpopulation, might be more susceptible to specific drugs compared to EVs from normal cells, thereby creating a dosing window for cancer therapy [113].

**Table 1:** Main anti-cancer EV-based therapeutic approaches.

EV-Based Approach	Specific Strategy	Example	References	Issues
**EV biology** **impairment**	Inhibition of EV release	GTPase and nSMase2 targeting	[114,115,116,117]	Specific targeting of cancer EV pathways
	Targeting on route	Affinity hemodialysis	[118]	
	Inhibition of EV uptake	Use of Heparin or Reserpin	[110,119,120]	
**Cancer** **immunotherapy**	Activation of immune response against tumor antigens	Dendritic cell-derived EVs	[121,122]	Low efficiency Safety
		Tumor-derived EVs	[121]	
**Native EV** **administration**	EVs from different sources	Mesenchymal stem cells	[123,124,125,126,127]	EV massive production and quality control EV stability, safety, administration and biodistribution
		Plants	[128,129,130]	
**EVs as** **drug-delivery** **systems**	Loading of natural compounds	Curcumin, traditional Chinese medicine ingredients	[131]	EV massive production and quality control EV engineering EV stability, safety, administration and biodistribution
	Loading of drugs	Chemotherapeutics	[132,133]	
	Loading of molecules targeting oncogenes or oncoproteins	siRNA	[134]	

Regarding EV production, some studies attempted to inhibit vesiculation by targeting Rab GTPase family components regulating vesicle exocytosis. In particular, Rab27a knockdown affected EV-mediated neutrophil mobilization, resulting in reduced tumor growth and decreased lung metastasis dissemination [114]. Moreover, the neutral sphingomyelinase 2 (nSMase2) involved in exosome biogenesis was identified as a key anti-cancer target. In fact, nSMase2 is more abundant in cancer cells compared to non-cancer cells, and its higher levels correlate with an increase in exosome formation and metastatic potential [135]. Recent studies identified GW4869, a chemical inhibitor of nSMase2, and Tipifarnib, a farnesyl transferase inhibitor downregulating nSMase2 and Rab27a, as new chemicals able to target exosome biogenesis [115,116]. In mouse models of colorectal cancers, a GW4869 intratumor injection was effective in counteracting tumor growth [117]. Natural compounds, such as the antifungal agent ketoconazole, were also identified as inhibitors of TDEV production [116]. Natural substances might have a better safety profile and less off-target and side effects, and thus are preferable to adopt in therapeutic approaches. EV release is also a well-established means through which cancer cells remove chemotherapeutics. Thus, targeting tumor EV biogenesis can be also adopted as a successful strategy to reduce chemoresistance. In this respect, different substances, including indomethacin, bisindolylmaleimide-I, chloramidine, calpeptin, and cannabidiol have been shown to enhance tumor response to chemotherapy through their proposed effect on blocking EV production, thereby increasing drug retention within tumor cells [136,137,138,139,140].

After being released, EVs can also travel long distances, for example, thorough circulation, paving the way to the possibility of targeting them “en route”. In this respect, affinity hemodialysis was proposed as a promising tool for preventing cancer metastasis and inhibiting EV-mediated immunosuppressive effects by selectively depleting circulating EVs released from various types of cancer cells [118]. A phase I clinical trial (NCT04453046) has been recently concluded (no results are available at the moment) to evaluate the efficacy and efficiency of using affinity hemodialysis by using hemopurifier specialized devices in combination with pembrolizumab in patients with advanced and/or metastatic squamous head and neck carcinoma [113].

Finally, TDEV uptake by normal niche cells at distant sites is a key mechanism in metastasis formation by adapting these cells to facilitate TDEV internalization and promoting the establishment of a pre-metastatic niche. Key players for cellular EV uptake are surface heparan sulfate proteoglycans (HSPGs), which are very heterogeneous across different tissue and cancer types [141]. In glioma cells, EV uptake is significantly impaired by the inhibition of HSPG biosynthesis, the enzymatic depletion of cell-membrane HSPGs and the use of free heparin and other heparan sulfate mimetics in a dose-dependent manner [87,119]. Heparin is able to block *in vivo* EV uptake and EV-mediated effects, as shown by studies on breast cancer with mouse models [120]. Reserpine, identified as an anti-hypertensive drug, was also found to inhibit TDEV uptake by normal cells, thereby blocking pre-metastatic niche formation and suppressing metastasis [110]. Noteworthily, heparins, both unfractionated and low-molecular-weight, were primarily used as anticoagulants, and then they were also found to be effective in slowing down the development of some types of cancer. Heparin was also hypothesized to suppress microvesiculation by mediating an attractive interaction between phospholipidic membranes. As the number of EVs isolated from peripheral blood was found to increase in patients with hypercoagulable states as well as in cancer patients, the suppression of microvesiculation by heparin might also account for both its anticoagulant and antitumoral effect [142]. Interestingly, all these findings point out promising cancer therapeutic strategies based on the repurposing of compounds that have been already clinically approved for other indications, to block EV-mediated tumor-promoting effects, in particular cancer metastasis. However, targeting EVs as a whole leads to a lack of cancer specificity. Instead, defining and targeting relevant tumor-associated EV cargo molecules may allow for a more specific and functionally defined approach [17]. Then, different intervention strategies would be possible, based on targeting the mechanisms controlling the sorting in the producing cells as well as the uptake or the downstream signaling in the recipient cells of specific TDEV-associated oncogenic molecules. Specific TDEV targeting still requires a detailed study of the composition of TDEV cargo (proteomic, lipidomic, metabolomic, genetic signature) and the associated functional mechanisms to identify the more promising cancer therapeutic targets.

### 5.2. Extracellular Vesicles as Therapeutic Particles

Completely different cancer-targeting approaches rely on the intrinsic therapeutic properties of native EVs or on the harnessing of EVs as nano-vehicles for the delivery of exogenous therapeutic molecules (Table 1). This is currently a very active research area, in which both academy and industry are strongly engaged, with many challenges concerning EV massive production and isolation, their detailed characterization and downstream manipulation [121,143].

Several *in vivo* studies have demonstrated that native EVs from various sources, including mesenchymal stem cells (MSCs), dendritic cells (DCs), and cells from plasma have cancer therapeutic potential [143]. In particular, EVs from DCs have been used to develop cancer vaccines and enhance immune responses in cancer patients. However, even though this approach gave promising results in pre-clinical studies, unfortunately, it showed modest T-cell activation in clinical trials involving melanoma and non-small-cell lung cancer patients, highlighting the need to increase DC-EV activity for successful immunotherapy [18,121,122]. Actually, EVs derived from MSCs are the best characterized type of EVs, but, in cancer, they have been described as a double-edged sword because of their capacity to both counteract and promote cancer progression [121]. The different behavior can be partially due to the heterogeneity of the originating MSC populations themselves, depending in turn on the source (bone marrow, cord blood, adipose tissue, dental pulp), the activation state and the external milieu, including extracellular matrix composition and stiffness and several physicochemical features, such as low/high pH, hyperoxia/hypoxia/anoxia, and low/high ionic gradients [144]. However, MSC-EVs originating from either murine or human bone marrow do suppress *in vitro* and *in vivo* angiogenesis by downmodulating *VEGF* expression in breast cancer cells [123,124]. Moreover, MSC-EVs from human bone marrow induced breast cancer cells to progressively dedifferentiate and survive as cancer stem cells in a dormancy-like state within the bone marrow [125]. Many antitumorigenic effects for MSC-derived EVs have also been reported on glioma, myeloma, prostate and lung cancer, mainly mediated by specific EV-associated miRNAs [126].

Finally, EVs have been exploited as novel platforms for drug delivery-based therapies (Table 1). Besides the biocompatibility and low immunogenicity, the high plasticity, loading ability, and genetic manipulation capability of EVs have made them the ideal nanoparticles for the delivery of therapeutic molecules. EVs may transport anti-cancer compounds to target cells, including drugs, proteins, aptamers and nucleic acids, mainly microRNAs and small interfering RNAs, prolonging their half-life and stabilizing their properties. Multiple loading strategies have been developed, primarily subdivided into endogenous or indirect methods, based on manipulating the parental cells, and exogenous or direct ones, based on the direct manipulation of EVs after isolation [19]. Several clinical trials are ongoing involving MSC-derived EVs, both unmodified and loaded with therapeutic molecules for various antitumorigenic applications, including the treatment of advanced colorectal cancer, pancreatic cancer, and breast cancer [127]. The finding that EVs are not bound to intraspecies interactions but are capable of interspecies and even interkingdom communication has once again revolutionized the EV field, opening new research directions. An increasing number of natural sources alternative to mammalian cells, which are possibly more economically viable and sustainable, are currently being investigated, including plants, bovine milk, bacteria, fungi, parasites, and microalgae [145,146,147,148,149]. In particular, EVs derived from plants (i.e., ginseng, citrus-limon, asparagus), both native and engineered, have been proposed as promising tools for anti-tumor therapy, with negligible side effects [18,128,129,130] Clinical trials (i.e., NCT01294072) involving plant-derived EVs for the treatment of cancer patients are ongoing [150].

Besides natural cell-derived EVs, another type of nanoparticles has recently taken the stage: bioinspired and biomimetic EV-like nanovesicles. These nanoparticles can be produced by multiple strategies and may have similar physicochemical properties to those of natural EVs, such as size, morphology, and even proteomic and lipidomic profiles. The most promising features of biomimetic nanoparticles regard production as well as modification procedures, which can be easier and more efficient, allowing for the high yield, homogeneity, and purity of the final samples, together with an improved targeting and drug-delivery efficiency [121].

## 6. Exploitation of Tumor-Derived Extracellular Vesicles in Anti-Cancer Therapies

TDEVs can not only be considered as a target for cancer therapy but can also be used for the development of anti-tumor therapeutic strategies. First of all, due to the presence of tumor antigens on the membranes, TDEVs were used to develop cancer immunotherapies (Table 1) [121]. TDEVs were used to activate dendritic cells *in vitro*, which, when injected *in vivo*, were able to stimulate the host immune system by boosting T-cell expansion and function [151]. However, TDEV participation in almost all aspects of tumor progression and development hinders their direct use as safe cell-free cancer vaccines [121].

In recent years, the exploitation of TDEVs as drug-delivery systems has received considerable attention. In fact, TDEVs are easily taken up by cancer cells *in vivo*, especially by the ones resembling the tumor cells of origin, showing high tumor-targeting and permeability activity [152]. Actually, the beneficial use of TDEVs for anti-cancer drug delivery has been demonstrated in several studies. The encapsulation of the monomeric active ingredients of traditional Chinese medicine inside TDEVs was efficient in improving their therapeutic effect [131]. The loading of chemotherapeutic and other biological drugs into TDEVs was also shown to enhance their curative effect [132,133]. Purified EVs from MCF-7 breast cancer cells were loaded with small interfering RNAs, miRNA, and single-stranded DNA oligonucleotides, and they were able to induce anti-cancer features by silencing *HER2* genes in recipient cells [134]. Some clinical trials using EVs from tumor cells for delivering drugs (e.g., NCT02657460 and NCT01854866) are ongoing [16], though their results are still not available. On the other hand, TDEVs might transport oncogenic cargo and eventually be able to promote tumor proliferation and metastasis formation. For this reason, the potential risks to human safety need to be carefully considered before starting clinical trials.

Noteworthily, TDEVs have been recently shown to have an antitumoral effect *per se*, paving the way for their possible use as natural anti-cancer therapeutics. In fact, EVs derived from NTERA2 teratocarcinoma cells are able to act on GBM cells, inducing a remarkable inhibitory effect on tumor cell migration, without inducing undesirable effects such as increased tumor cell proliferation or chemotherapy resistance [153]. Teratocarcinoma is a common type of testicular germ cell tumor mostly formed of embryonal carcinoma stem cells. After retinoic acid (RA) exposure, teratocarcinoma cells can differentiate into postmitotic neurons and glia, thus showing neural stem/progenitor cell properties [154,155]. NTERA2 cells or NTERA2-derived neural precursors partially differentiated with RA showed GBM tropism *in vitro* and *in vivo*, respectively, and were proposed as promising drug-delivery cellular systems for targeting GBM cells [156,157]. However, as teratocarcinoma is considered a highly aggressive cancer, the anti-migratory effect mediated by native NTERA2-derived EVs, without any drug loading, is quite unexpected. Even more unexpectedly, this effect seems to be mediated by the onco-developmental factor Cripto [153].

Cripto is a membrane-bound glycosylphosphatidyl inositol-anchored protein that can act as a cofactor for TGFβ and its family members, including Nodal and Activin, and it is also released from the plasma membrane as a soluble protein [158,159,160]. Cripto has a key role in early embryo development, for mesoderm formation and anterior-posterior patterning [161,162,163,164], and also in tumor progression. An antitumoral effect for Cripto was previously proposed by a study on colon tumor induction in mouse models, showing that *Cripto* haploinsufficiency increased colon tumorigenesis [165]. However, most studies pointed instead to an oncogenic role of Cripto, associated with increased cancer features, including metastasis induction, and a worse patient prognosis [158,166,167,168,169]. *Cripto* overexpression, and, more interestingly, the soluble Cripto form alone or the induction of Cripto shedding from the cell membrane, were found to stimulate the migration of different types of cells, including mammary epithelial cells, human umbilical vein endothelial cells and GBM cells [170,171,172,173]. The migration impairment of GBM cells through Cripto-EVs, instead, may point to a high grade of complexity for the fine-tuning of Cripto localization, route and consequent function [153].

**Figure 4:**
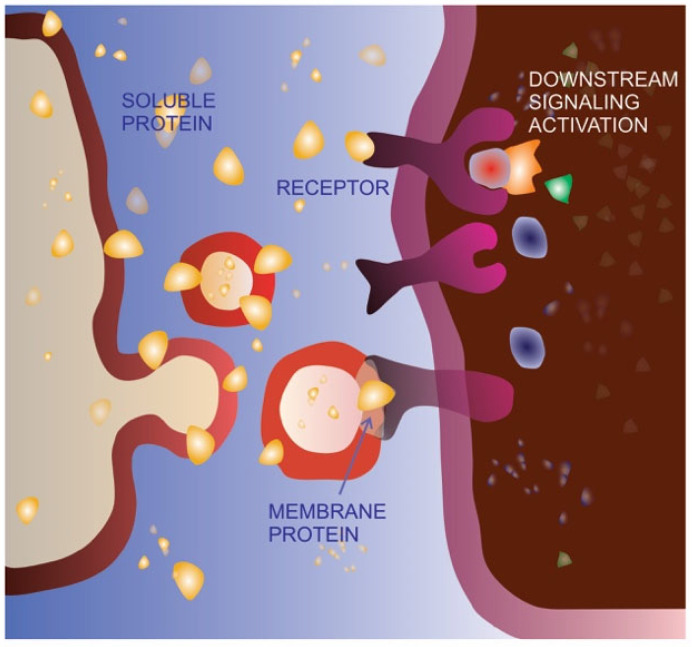
Extracellular vesicle-mediated delivery as an alternative signaling route. The same protein can be bound to the cell membrane, released in the extracellular space as a soluble molecule or delivered on the membrane of extracellular vesicles (EVs). As a soluble ligand, the protein can recognize, bind and activate its receptor on the membrane of the target cell. The EV-delivered molecule is able to bind the receptor on the target cell, but does not activate the same signaling pathway, possibly acting as a dominant-negative form.

Cell migration is an extremely finely regulated process. The same stimulus can have a different effect on cells depending on the cellular context (cell and cancer type) as well as the stimulus concentration. It has been clearly demonstrated that the key EMT inducer TGFβ is able to promote an incomplete or hybrid EMT phenotype in specific cell types, characterized by the acquisition of mesenchymal features, metastasis promotion and concomitant maintenance of cell–cell adhesion [174,175,176]. In specific contexts, TGFβ is also able to act in a very opposite way, behaving as a tumor suppressor [177,178]. Moreover, platelet-derived growth factors at low concentrations can promote cell migration, whereas at high concentrations, they may induce proliferation [179]. Both molecules have been found to be associated with EVs, in the soluble form as well as in the precursor form bound to the membrane [180,181], allowing us to hypothesize that encapsulation inside EVs and/or exposition on the EV surface might be an additional mechanism for regulating the spreading and activity of soluble and/or membrane-bound signaling molecules. In other words, EV sorting and delivery might be a physiological and/or pathological alternative route for key signaling molecules, modulating their final impact on cancer development and progression, as schematized in Figure 4.

The rationale for tumor cells producing EVs with anti-migratory effects might be to target circulating cancer cells and promote a MET transition, with their exit from circulation and homing in the forming metastatic sites. Besides their intrinsic ability to target specific tumors and/or tumor microenvironments, unmodified TDEVs might concomitantly be able to deliver potential endogenous migration inhibitors and/or interfere with the migration signaling pathways of the target cell. The idea that, due to these features, specific subsets of EVs released by tumor cells might be enriched and used *per se* in anti-cancer therapeutic strategies, is extremely disruptive. Indeed, because of the low yield of commonly used loading procedures, the necessity of post-modification separation and quality control, the introduction of exogenous material into the vesicles is definitely a bottleneck in the pipeline for the development of EV-based therapies. Finally, even in the case of the delivery of cancer therapeutic molecules, anti-migratory TDEVs might be the carriers of choice as they already have intrinsic anti-tumor properties, which could favor the action of the exogenous loaded molecule.

## 7. Potentialities and Pitfalls for EV Exploitation

Recent decades have seen an expanding interest in the EV field and in the development of EV-based therapeutics. The increasing number of research studies highlighted the complexity of dealing with EVs, due to their extreme heterogeneity as well as the heterogeneity of the methodologies adopted for their isolation and characterization, together with poor quality assurance and control of the EV samples obtained. Nowadays, data reliability and reproducibility have been recognized as main issues in life sciences and in particular inside the EV community [182,183,184,185,186,187,188,189]. Since the International Society for Extracellular Vesicles (ISEV) was founded in 2012, the EV community has been committed in a strong effort to identifying and providing guidelines for an increasing number of aspects in EV research, such as sample collection, processing, isolation, and characterization [190,191,192,193,194,195]. The application of specific quality-management tools or the adoption of a customized quality-management system proved also useful for the optimization and standardization of EV-related processes, together with the assessment and management of process failures and related risks [185,196,197,198,199,200]. However, despite the great effort and the positive examples provided, a great number of studies on EVs do not yet take full advantage of the guidelines identified, or the available data- and quality-management tools.

Moreover, EV exploitation for the therapy of cancers as well as other diseases carries with it other fundamental issues related to the massive production of EVs to be used in clinics. These issues include the identification of a suitable and reliable EV source, the scaling-up of the production, the identification of high-throughput isolation technologies, and last but not least, EV engineering with therapeutic molecules [201]. In the case of engineered EVs, besides the loaded molecules, EVs also confer their own biological activity based on their native cargo content. These dual modes of action need to be carefully considered for clinical applications [126]. Noteworthily, all the processes must be set up and put in place while ensuring compliance with good manufacturing practice (GMP).

Finally, the pharmacokinetic properties of drug-delivery systems are also a key issue for delivering medication where and when it is required. Several studies have been undertaken to assess EV biodistribution. EVs from different sources are mainly detected in the spleen, liver, lungs, kidneys and gastrointestinal tract. Notably, EVs were also detected in the brain, and their ability to penetrate the blood–brain barrier is one of the main advantages provided by EVs as delivery vehicles [202,203,204,205]. EV pharmacokinetics, however, may be affected by the source, the isolation method used, the loading procedure and cargo, as well as the administration route and dose. The assessment of the amount of EVs to administer to cancer patients, the application form (local or systemic) and the frequency of repetitive vesicle injections require the careful validation of the results on EV stability, half-life and biodistribution, together with therapeutic biosafety and potency assays for comparable bioactivities, dosage, and pharmacokinetics. In this respect, data deriving from the ongoing clinical trials will be fundamental. Moreover, several issues related to pre-clinical tests and settings as well as regulation for EV therapeutics (although they could belong to the pharmaceutical class of biologicals) need to be further addressed in order to reach the desired ambitious goals related to pharmaceutical EV manufacturing and routine clinical applications.

## 8. Conclusions and Perspectives

Cancer cell migration is a hallmark in cancer pathogenesis, whose targeting is crucial to prevent tumor spreading and metastasis. Recent advances indicate that cells communicate by means of nano-sized particles called EVs, which have pleiotropic effects during cancer progression and metastasis development. EVs rose to prominence and will continue to be a focus of attention in the future for understanding the mechanisms of cancer cell migration and developing innovative anti-tumor therapeutic approaches. Several strategies are ongoing, including the inhibition of TDEV secretion, transportation or uptake as well as the exploitation of EVs from different sources for delivering specific therapeutic molecules to cancer cells. In this scenario, paradoxically, TDEVs themselves might be promising therapeutic agents for targeting cancer cells and counteracting cancer features by delivering exogenous or even endogenous cargo. An EV shuttle might be an additional mechanism to regulate the spreading and the activity of key soluble and/or membrane-bound signaling molecules, modulating their effect on target cells and the final impact on cancer development and progression. Translating these research studies into successful anti-cancer therapies is an ambitious and desirable goal that will require a strong commitment from researchers and the further implementation of a multidisciplinary approach, integrating biological, physical, chemical, mathematical, pre-clinical and clinical data in a standardized and quality-controlled environment.
